# Quality of life during a randomized trial of a therapeutic-workplace intervention for opioid use disorder: Web-based mobile assessments reveal effects of drug abstinence and access to paid work

**DOI:** 10.1016/j.dadr.2021.100011

**Published:** 2021-12-04

**Authors:** Jeremiah W. Bertz, Kirsten E. Smith, Leigh V. Panlilio, Samuel W. Stull, David Reamer, Marie-Louise Murville, Michael Sullivan, August F. Holtyn, Forrest Toegel, David H. Epstein, Karran A. Phillips, Kenzie L. Preston

**Affiliations:** a Intramural Research Program, National Institute on Drug Abuse, 251 Bayview Blvd., Suite 200, Baltimore, MD 21224, United States; b Delight Me Inc., Washington, DC, United States; c Department of Psychiatry and Behavioral Sciences, Johns Hopkins University School of Medicine, Baltimore, MD, United States

**Keywords:** Contingency management, Opioid use disorder, Cocaine, Urinalysis, Smartphone, Quality of life

## Abstract

**Background:**

Employment and improved quality of life (QOL) are, separately, valued outcomes of substance use disorder (SUD) treatment. It is also important to understand QOL changes caused by employment itself; therefore, we assessed QOL during a randomized trial of a contingency-management-based Therapeutic Workplace for people with opioid use disorder.

**Methods:**

For 12 weeks, participants (*n* = 61) responded to QOL questionnaires in a mobile web app accessed with study-issued smartphones. At enrollment, participants were randomized to work in the Therapeutic Workplace immediately (immediate work group, IWG) or after a 3-week waitlist delay (delayed work group, DWG). Once both groups could work, wage-resetting contingencies were introduced for their opiate- and cocaine-urinalysis. Data were analyzed by (1) access to work with and without contingencies and (2) overall urinalysis-verified opiate- and cocaine-abstinence.

**Results:**

DWG and/or IWG reported improvements in several QOL areas (sleep, transportation, recreation); however, they also reported increased money-related difficulties and less time spent with friends/family. These changes did not coincide with DWG's work access, but some (more sleep, money-related difficulties) coincided with the urinalysis contingencies. Greater opiate- and/or cocaine-abstinence was also associated with several improvements: sleep, paying bills, time spent with friends/family, and exercising. Surprisingly, intermediate cocaine abstinence was associated with reductions in work-capacity satisfaction and recreation.

**Conclusions:**

Participants reported complex QOL differences during their experimental employment and associated with drug abstinence. Future work should help participants address issues that may be relevant to employment generally (e.g., time with friends/family) or contingency management specifically (e.g., money-related issues for non-abstinent participants).

## Highlights

• Quality of life (QOL) is a useful outcome in opioid use disorder (OUD) treatment.
• We used a mobile web app to assess QOL during contingency management for OUD.
• Participants reported increases in several QOL areas, but also in money problems.
• Greater drug abstinence was associated with better QOL in several areas.
• Mobile QOL measures could help in assessing different treatment responses.

## 1 Introduction

Quality of life (QOL) measurements provide insight into a person's daily life, life satisfaction, and functioning relative to others, either globally or related to specific health conditions and their treatment (e.g., Ferrell et al., 1995; Kluivers et al., 2008; Lohr et al., 2002; Muldoon et al., 1998; Schalock, 2004), including for substance use disorders (SUDs) (Bray et al., 2017; Donovan et al., 2005; Laudet, 2011; Pasareanu et al., 2015; Rudolf and Watts, 2002; Tracy et al., 2012). People with SUDs have been found to have lower QOL compared to healthy populations, with low QOL potentially contributing to continued drug use (Dingle et al., 2015; Karow et al., 2011; Kelly et al., 2018; Kiluk et al., 2019; Laudet et al., 2009; Maremmani et al., 2007; Smith and Larson, 2003; Stevanovic et al., 2015). Conversely, SUD treatment has been associated with improvements in QOL and/or related measures of psychosocial functioning (Giacomuzzi et al., 2003, 2005; Maremmani et al., 2007; Pinto et al., 2010; Vigezzi et al., 2006; see also Ponizovsky and Grinshpoon 2007). Therefore, QOL improvements may not only influence “primary” SUD treatment outcomes such as retention and reductions in drug use (e.g., Best et al., 2013; see also Dennis et al., 2020), but also be functional outcomes of treatment themselves, or encompass lifestyle factors supporting or reflecting changes in drug use (Bonomi et al., 2000; Felce and Perry, 1995; Laudet et al., 2009; Manning et al., 2019; Muller et al., 2016; Tiffany et al., 2012).

Considering these lifestyle factors, relationships among SUD treatment, employment, and QOL may be particularly noteworthy. Employment has long been recognized as an important “secondary” SUD treatment outcome (French et al., 2002; Ouimette et al., 1998; Richardson and Epp, 2016; Sacks et al., 1997; Vanderplasschen et al., 2013), and engaging in work or job-related activities (i.e., job-searching and -training activities) has been shown both to reduce employment problems and improve QOL among people with SUDs (Petry et al., 2014; see also De Maeyer et al. 2011). Several different kinds of intervention have been proposed to improve the employment outcomes of people with SUDs (reviewed, e.g., by Magura et al., 2004; Magura and Marshall, 2020). Among these, the Therapeutic Workplace integrates contingency management with employment, making access to paid vocational training and other work activities contingent on urinalysis-verified drug abstinence (Silverman et al., 2016; Wong and Silverman 2007). Participants initially are given access to computerized job-skills training activities in a laboratory workplace setting. Over the course of the intervention, participants’ ability to enter the workplace or the stipend they receive for completing workplace activities (conceived of as a “wage” for study work) is gradually made contingent on their providing drug-negative urine samples, with positive or missing samples preventing access or producing temporary wage reductions. These contingencies have been shown to improve retention in the Therapeutic Workplace and reduce drug use (reviewed by Silverman et al., 2016; Wong and Silverman 2007), and Therapeutic Workplace training followed by a program of abstinence-contingent wage supplementation has been shown to improve participants’ community employment (Holtyn et al., 2020).

Contingency management, which continues to be a key behavioral intervention for SUDs generally (e.g., Bentzley et al., 2021), has been implemented in diverse settings, and contingency management for illicit drug use has been associated with improved QOL outside of the Therapeutic Workplace. For example, Petry et al. (2007; see also Andrade et al., 2012) found the addition of contingency management to standard-of-care intensive outpatient treatment improved QOL in people who use cocaine, and Epstein et al. (2009) found improved QOL in people with opioid use disorder (OUD) receiving methadone and contingency management. However, there may also be particular considerations when implementing contingency management via the Therapeutic Workplace or other employment-related means. Paid work may improve several aspects of QOL (e.g., money-related issues and other issues such as transportation that could be improved with increased income; social isolation through interactions with coworkers), but other QOL aspects may be adversely impacted by “work-life balance” issues (e.g., recreation, social events/activities unrelated to work) or work-related stress (for more on the possible psychosocial benefits of paid work, see Henkel 2011; Monaghan and Wincup 2013; Platt 1995).

Although QOL measurements in people with SUDs have varied in instrument type and target subpopulation (e.g., people who inject drugs, people in recovery vs. with active use) (Brogly et al., 2003; González-Saiz et al., 2009; Hubley and Palepu, 2007; Laudet, 2011; Strada et al., 2017; Tiffany et al., 2012; Wan et al., 2011), QOL questionnaires generally assess wellbeing, i.e., perceptions of one's life conditions and satisfaction (Gill and Feinstein, 1994; Ware, 1976), across multiple domains, including physical, material, social, emotional, and development and activity (Felce and Perry, 1995). Among people with SUDs and other health conditions, multiple facets of QOL—including physical and emotional health, accessible healthcare, accessible transportation, financial stability, spirituality, social support and satisfaction, and the capacity to work and maintain employment—have been identified as having potential clinical utility (Brady et al., 1999; De Maeyer et al., 2010; Laudet et al., 2006; Morgan et al., 2003; Muller et al., 2016). These different QOL domains and their component elements are often dynamic, changing over time and with different life experiences (Allison et al., 1997; Donlin et al., 2008; Newbern et al., 1999). Therefore, to understand QOL changes in relation to particular treatment events or outcomes, it is important to track QOL in detail over time. Changes among the individual aspects of QOL may be lost in summary measures or global scores (cf., Petry et al., 2007). Likewise, the longer inter-assessment intervals used in previous studies of contingency management and QOL (6 weeks to 2–3 months, EÉk et al., 2020; Petry et al., 2007) may be well suited to track longer-term or more enduring changes, but they may not be ideally suited to capture changes related to specific life events or, for research participants, study conditions (but, see also Epstein et al., 2009).

The aims of this study were (1) to assess the feasibility of measuring several core aspects of QOL repeatedly (i.e., daily or weekly) using study-issued smartphones and mobile web-based questionnaires during a Therapeutic Workplace intervention for people with opioid use disorder, and (2) to explore differences in these aspects over time in relation to participants’ Therapeutic Workplace access and abstinence-reinforcement contingencies, as well as their overall level of verified drug abstinence. We sought to identify whether decreases (or increases) in specific components of participants’ QOL are associated with particular treatment events and outcomes.

## 2 Methods

### 2.1 Setting and participants

This work was conducted during a longer study, referred to here as the Workplace Study, of the effects of abstinence-contingent wage supplementation on employment and drug abstinence (ClinicalTrials.gov NCT02487745; Holtyn et al., 2020, Toegel et al., 2020) by the Johns Hopkins University Center for Learning and Health (CLH) in Baltimore, MD, USA. All study procedures were approved by the Johns Hopkins Medicine IRB, and all participants provided prior written informed consent.

Data for the present analyses were collected between May 2017 and July 2018, when all participants were engaged in 3 months of abstinence-initiation and job-skills training (computer-based skills-training and educational activities) performed at the CLH Therapeutic Workplace as part of the Workplace Study. This study concluded when the Workplace Study completed its participant enrollment. Baseline participant characteristics were collected with the ASI-Lite (Cacciola et al., 2007). For the Workplace Study, inclusion criteria were: age 18 years or older, residence in or near Baltimore City, current unemployment with self-reported interest in gaining employment, current enrollment in or eligibility for methadone or buprenorphine maintenance treatment, and an opioid-positive urine sample. Exclusion criteria were: physical limitations that prevented typing, current suicidal or homicidal ideation, active psychotic disorder symptoms, or current imprisonment. For the participants who provided QOL data, there were no inclusion/exclusion criteria concerning the use of drugs other than opioids.

All Workplace Study participants enrolled during this time (*n* = 67) were offered a smartphone (Samsung Galaxy Grand Prime or Samsung Galaxy On5 models; Samsung Telecommunications, Suwon, South Korea) to complete the web-based QOL questionnaires (Section 2.3). Separately, participants used a different app on the smartphone to provide ecological momentary assessment (EMA) data, and the QOL questionnaires were administered in addition to the EMA questionnaires. Details of the EMA data collection and analyses will be described elsewhere (manuscript forthcoming). Presently, we are focusing only on the QOL data; however, in considering participants’ study completion/non-completion, it is important to note that noncompliance with EMA data collection resulted in termination of both EMA and QOL data collection because the same device was used for both. Participants’ smartphone-based tasks could also be terminated if the smartphone was lost or damaged, or if participants did not have their (operational) smartphone with them on 12 or more days at the Therapeutic Workplace. Noncompliance only with the QOL questionnaires (Section 2.3.2) did not result in study termination. In all cases, termination of participants’ smartphone-based tasks was separate from their attending the Therapeutic Workplace itself (i.e., participants who had their smartphone-based tasks terminated could continue to attend the Therapeutic Workplace without change); however, smartphone-based data collection was also terminated for any participant discharged from the larger Workplace Study.

### 2.2 Therapeutic Workplace procedures

This study was designed to assess changes associated with participants’ ability to access paid work in the Therapeutic Workplace and, once they could access paid work, changes associated with the introduction of urinalysis contingencies that reset their experimental “wages” to reinforce abstinence from opiates and cocaine. These conditions were implemented as follows (see supplementary Figure S1 for a schematic experimental timeline).

Upon enrollment, participants were randomized to the Immediate Work Group (IWG) or Delayed Work Group (DWG). Participants were randomly assigned (1:1) to one of the two study groups using a computerized urn randomization procedure (Wei and Lachin, 1988). Various staff members operated the randomization program, but the NIDA investigators who collected participants’ smartphone-based data were not involved in randomization. IWG could access paid work in the Therapeutic Workplace for up to 4 h each day Monday-Friday beginning on the day of their randomization. For DWG, a waitlist delay was in force for the first 3 weeks after randomization: DWG could not yet access paid work in the Therapeutic Workplace but was paid by the Therapeutic Workplace as though they had engaged in all paid work activities available to IWG. Both DWG and IWG were paid for smartphone-related tasks regardless of experimental phase (i.e., during and after the waitlist delay) and were asked to provide urine samples for drug screening (typically on Mondays/Wednesdays/Fridays) and to meet with investigators for smartphone-related tasks. After the waitlist delay, when both IWG and DWG could access paid work in the Therapeutic Workplace under identical conditions, wage-resetting contingencies were introduced in successive phases to reinforce participants’ abstinence, first from opiates and then from both opiates and cocaine. Wage resets (i.e., temporary reductions from $8.00/hour to $1.00/hour) were produced by drug-positive or missing urine samples (for more details see Toegel et al., 2020).

Participants’ time in the Therapeutic Workplace was, thus, divided into four phases: (Phase 1) the 3-week “Waitlist” phase when only IWG could access paid work, with no wage-resetting contingencies; (Phase 2) the 2-week “Induction” phase, when both IWG and DWG could access paid work, with no wage-resetting contingencies; (Phase 3) the 2-week “Opiate contingency” phase (Opi), when both IWG and DWG could access paid work, and a wage-resetting contingency for opiate-positive, but not cocaine-positive, urinalysis was in place; and (Phase 4) the 7-week “Opiate + cocaine contingency” phase (Opi+Coc), when both IWG and DWG could access paid work, and there were wage-resetting contingencies for opiate-positive and/or cocaine-positive urinalysis. To ease training and improve compliance, EMA data collection and QOL questionnaires were introduced sequentially to participants: smartphones were issued and EMA training conducted one week after randomization, and QOL questionnaires were introduced one week after EMA training. Thus, participants provided QOL data from the final week of Waitlist onward.

### 2.3 QOL questionnaires

QOL data were collected via the web-based Delight Me platform (www.delightme.com; Delight Me Inc., Washington, DC, USA). Participants were given access to the Delight Me web app and trained to respond to daily and weekly questionnaires developed to measure facets of participants’ life in domains consistent with QOL.

Participants accessed the Delight Me web app using their study-issued smartphones. Participants were scheduled to meet with study staff for training on the Delight Me web app one week after they received their study smartphones and began EMA data collection. Thereafter, participants continued to provide EMA data as they had been trained, as well as responding to QOL questionnaires.

Prior to training, study staff created Delight Me accounts for participants using their study-issued telephone numbers and a distinct participant code number; no personally identifiable information was provided to Delight Me Inc.. Beginning with the day of their training, upon logging in to their accounts, participants were automatically presented with the questions they were scheduled to answer that day.

#### 2.3.1 Daily and weekly questionnaires

Questionnaires were developed by study staff and custom-programmed by Delight Me for delivery. Participants received 2 questions daily about their sleep: duration and the extent to which they felt rested from sleep. Once per week, participants received a longer questionnaire containing the same 2 questions on sleep plus 28 questions on the following topics: (1) “health and well-being,” (2) “money and budget,” (3) “mobility and transportation,” (4) “social support: friends and family members,” and (5) “recreation.” Table 1 presents the full list of questions and response options. These topics were selected broadly to include domains of daily life functioning that could be improved by access to paid work (e.g., money and budget, with impacts on other areas from increased income); be adversely impacted by access to paid work (e.g., recreation, as time is spent at work vs. leisure); and/or present potential obstacles to working (e.g., transportation or health problems). The QOL questionnaire that we developed for this study was not pre-tested or validated separately in a prior study. However, the topics that we included are known to be important to people in OUD treatment, as we have seen in clinical experience providing treatment for OUD in the NIDA IRP Archway Clinic and described in previous papers (e.g., on EMA-reported “daily hassles,” Preston et al., 2018; on EMA-reported activities, social contexts, and drug use, Epstein and Preston, 2010). Assessing QOL was an exploratory objective of the Workplace Study, and each question was analyzed as an exploratory endpoint, as described below (Section 2.4).

**Table 1:** Full text of the quality of life questions administered via smartphone.

Topic	Question	Response options
*Health and well-being*	About how many hours of sleep did you get last night?	Fill-in-the-blank (hours)
	Do you feel rested?	Yes/No
	Do you have any unmet health needs?	Physical, Emotional, Both, Neither
	Did you seek or receive any healthcare services this week?	Physical, Emotional, Both, Neither
	How satisfied were you with the physical healthcare you received?	5-point scale (1 = Very dissatisfied, 5 = Very satisfied), or N/A
	How satisfied were you with the emotional health care you received?	5-point scale (1 = Very dissatisfied, 5 = Very satisfied), or N/A
	How much did pain interfere with your activities this week?	5-point scale (1 = Not at all, 5 = An extreme amount)
	How satisfied are you with your capacity for work?	5-point scale (1 = Very dissatisfied, 5 = Very satisfied)
*Money and budget*	Did you have enough money to meet your needs this week?	Yes/No
	Did all your bills get paid?	Yes/No
	Do you have any money set aside for emergencies?	Yes/No
	Was money a problem for you this week?	Yes/No
*Mobility and transportation*	Was transportation a problem for you this week?	Yes/No
	Were you able to get everywhere you needed to this week?	Yes/No
	What mode of transportation did you use the most this week?	Walk, MTA, I drove, Others drove (someone gave me a ride), Bike/scooter, Paid other to drive (like a cab or "hack")
	What mode of transportation did you use second most this week?	Walk, MTA, I drove, Others drove (someone gave me a ride), Bike/scooter, Paid other to drive (like a cab or "hack")
*Social support: friends and family members*	About how much time did you spend with friends and family?	Fill-in-the-blank (hours)
	About how much time did you spend with non-drug-using friends and family?	Fill-in-the-blank (hours)
	How satisfied are you with time spent with friends and family?	5-point scale (1 = Very dissatisfied, 5 = Very satisfied), or N/A
	How satisfied are you with time spent with non-drug-using friends and family?	5-point scale (1 = Very dissatisfied, 5 = Very satisfied), or N/A
*Recreation*	Did you do any enjoyable activities this week?	Yes/No
	How many times did you do an enjoyable activity this week?	Fill-in-the-blank (number)
	Was the activity related to…	
	a personal hobby?	Yes/No
	self-improvement?	Yes/No
	healthy eating?	Yes/No
	unhealthy eating?	Yes/No
	exercise?	Yes/No
	a social event?	Yes/No
	a spiritual or religious activity or event?	Yes/No
	other activity?	Yes/No + Fill-in-the-blank

Participants received automated text message alerts, including a hyperlink to the Delight Me website, to remind them to complete their scheduled questionnaire. Each day, participants who had not completed their scheduled questionnaire by then received a first message at 9:00 am and, if still not completed, a second message at 5:00 pm. Participants could access the questionnaire until midnight each day, when it “expired.”

#### 2.3.2 Compliance and remuneration

QOL data were reviewed with participants weekly. Participants were paid up to $10.00 per week for completing their questionnaires as follows: (1) $5.00 for completing the daily questionnaire on at least 4/7 days, and (2) $5.00 for completing at least 16/30 questions in the weekly questionnaire. These payments were made in addition to separate payments of up to $50.00 per week for EMA data collection, for a total of up to $60.00 per week for completing smartphone-based tasks. These payments for smartphone-based tasks were independent of participants’ wages earned in the Therapeutic Workplace.

### 2.4 Data analysis

Participant characteristics were compared using *chi*-square or Fisher's exact test for categorical variables and *t*-tests or Mann-Whitney *U* for continuous variables. Data from QOL questionnaires were analyzed as described below. To separate completers from non-completers, non-completers were defined as participants who had their QOL data collection terminated before the end of the Opi+Coc phase for any of the reasons listed above (Section 2.1). For all analyses, differences were considered significant when *p* < .05, two-tailed, with trends noted when *p* < .10. Participant characteristics were analyzed using SPSS Statistics Subscription (IBM; Armonk, NY, USA). QOL data were analyzed using SAS version 9.4 (SAS Institute; Cary, NC, USA) or R version 3.6 (R Core Team, 2018).

#### 2.4.1 Analysis 1: effects of access to paid work on QOL

The first set of analyses assessed effects of access to paid work in the Therapeutic Workplace by comparing IWG vs. DWG over time (i.e., across phases). To ensure that all participants contacted all 4 phases of the Therapeutic Workplace intervention, these analyses were conducted with the *n* = 34 completers.

For each continuous endpoint, a linear mixed model (SAS proc MIXED) was constructed including Group (IWG, DWG), Phase (Waitlist, Induction, Opi, Opi+Coc), and the Group X Phase interaction. Control terms for Sex (male, female); Race/ethnicity (white, non-white); vocational Skill (skilled, semi/unskilled; categorized by the Hollingshead index as collected by the ASI-Lite), and Age (years) were also included in all models. As several QOL aspects may show seasonal variations (e.g., sleep, Bertz et al., 2019; physical and mental health, Jia and Lubetkin, 2009; socializing, Simonsen et al., 2011), we also screened each endpoint for effects of astronomical season with a univariate model. For endpoints with univariate *p* < .20, season was also included in the final multivariate model: previous-night hours of sleep and the amount of time spent with friends and family who do not use drugs. Pairwise comparisons were performed *post hoc* within groups among phases or within phases between groups using *t*-tests with the adaptive Holm method (SAS proc MULTTEST) used to correct for multiple comparisons.

For each categorical endpoint, a generalized linear mixed model (SAS proc GLIMMIX) was constructed as for the continuous endpoints, except age was dichotomized (<45 years, ≥45 years) because models with either the individual years or 10-year bins did not converge. Season was included for the following endpoints: paying bills, money being a problem, transportation being a problem, engaging in a social event as recreation, and engaging in an “other” activity as recreation. Model-derived-means and odds ratios were determined according to SAS Usage Note 24,455 (SAS Institute 2021). Adjusted odds ratios are presented in all cases. For multiple pairwise comparisons, the STEPDOWN option was used in the ESTIMATE statement of proc GLIMMIX to produce Holm-corrected *p-*values (although the confidence intervals are still based on the Bonferroni correction, as this cannot be changed by the STEPDOWN option).

An autoregressive error structure was used in all models. Denominator degrees of freedom were calculated with the between-within approximation method. Predictors were treated as fixed, and a fixed intercept was used, because models with a random intercept did not converge.

Given the relatively large number of separate QOL endpoints analyzed, we corrected the *p*-values for the effects of Group and Phase in the final multivariate models using the adaptive False Discovery Rate (FDR) procedure (SAS proc MULTTEST; Benjamini and Hochberg 2000), and we considered pairwise differences only when omnibus *p*_FDR_ < 0.10. Calculating effects sizes in multilevel models is an area of emerging research and evolving guidelines (e.g., Lorah, 2018; Rights and Cole, 2018); for effects with *df*_numerator_ = 1, we calculated effect size as *r*_effect_ from *F* by the method of Rosnow et al. (2000).

#### 2.4.2 Analysis 2: associations between verified drug abstinence and QOL

The second set of analyses assessed associations between participants’ abstinence, as verified by their Therapeutic Workplace opiate and cocaine urinalysis results, and QOL. These analyses were conducted with all *n* = 61 participants with any QOL data.

Separately for opiates and cocaine, participants’ overall rate of verified abstinence was calculated as their percent negative urine samples (vs. positive or missing), and each participant's rate was classified as Low Abstinence (0.0–33.0% negative), Intermediate Abstinence (33.1–66.0% negative), or High Abstinence (66.1–100.0%). For opiates, the group sizes were: Low, *n* = 15; Intermediate, *n* = 12; High, *n* = 34. For cocaine, the group sizes were: Low, *n* = 27; Intermediate, *n* = 10; High, *n* = 24. One participant provided 100% urine samples negative for both opiates and cocaine, and 1 participant provided 0% urine samples negative for both opiates and cocaine. All other participants provided mixtures of negative samples, missing samples, and samples positive for opiates and/or cocaine. The cutoffs among abstinence rates used here are somewhat arbitrary, but they provide a manageable and clinically intuitive set of outcomes to explore, and the Low Abstinence category corresponds at least approximately to the standard that, in monitoring urinalysis, drug use on at least 25% of days represents treatment failure (Goldstein and Brown 1970), i.e., ≥ 2 positive samples per week from use approximately twice per week (1.75 days/week) with each use producing 1–2 positive urinalysis results depending on its exact timing.

Models were constructed as in Analysis 1, except each QOL endpoint was analyzed by Abstinence (Low, Intermediate, High), with Group and Phase included among the control terms. As in Analysis 1, we corrected the *p*-value for the effect of Abstinence in the multivariate models using the adaptive FDR procedure, and we considered pairwise differences only when omnibus *p*_FDR_ < 0.10.

#### 2.4.3 Analysis 3: nonmetric multidimensional scaling of all QOL items

Finally, we used an exploratory nonmetric multidimensional scaling (NMDS) procedure to assess and visualize relationships between pairs of QOL items and between each QOL item and the Therapeutic Workplace phase (Waitlist, Induction, Opi, Opi+Coc) in effect for each day the questionnaire was answered, using the same data as Analysis 1. NMDS is similar to principal components analysis, except NMDS does not assume continuous data or linear relationships (i.e., it can use any measure of association), and unlike principal components analysis, the solution (distance matrix) produced by NMDS is based on rank orders and numerical optimization, not eigenvalues. Compared to other methods for data reduction, NMDS may be particularly appropriate for representing relationships with relatively few dimensions (for more on NMDS see Hout et al., 2013; Rabinowitz 1975). Distance was defined as 1 minus the Spearman rank correlation coefficient for each pair of items. The Sammon NMDS procedure was applied (*R* package MASS) to all pairs to obtain scalings in two dimensions, and then all items were included in a two-dimensional plot.

## 3 Results

### 3.1 Participant characteristics

Table 2 presents participants’ demographic and baseline characteristics. Among the full sample of *n* = 61 participants who provided any QOL data, completers were significantly younger than non-completers. There were also trends for completers to have less non-employment income and more days receiving opioid agonist treatment (OAT) in the past month. All other differences between completers and non-completers and, among the *n* = 34 completers, between IWG and DWG were not significant.

**Table 2:** Participant characteristics, between-group differences for completers vs. non-completers, and differences between those randomized to the Immediate vs. Delayed Work Group among study completers^f^.

	Total enrolled, *n* = 61	Completers,*n* = 34 (55.7%)	Non-completers,*n* = 27 (44.3%)	*p*	Completers, *n* = 34	Immediate Work Group,*n* = 21 (61.8%)	Delayed Work Group,*n* = 13 (38.2%)	*p*^a^
*Age*	47.4 (11.5)	44.5 (10.6)	51.1 (11.8)	.016	44.5 (10.6)	43.9 (11.8)	45.4 (8.5)	.807
*Male*	62.3%	58.8%	66.7%	.717	56.8%	63.0%	50.0%	.308
*Race/ethnicity*				.553				.867
African American/Hispanic	60.6%	55.9%	66.7%		55.9%	52.4%	61.5%	
White	39.4%	44.1%	33.3%		44.1%	47.6%	38.5%	
*Married*	16.4%	14.7%	18.5%	.476	14.7%	14.3%	15.4%	1.00
*Currently on probation/parole*	11.5%	14.7%	7.4%	.374	14.7%	14.3%	15.4%	1.00
*Lifetime incarceration history*	85.2%	88.2%	77.8%	.315	88.2%	90.5%	84.6%	.627
Months incarcerated^b^	65.1 (86.3)	45.6 (45.7)	91.7 (117.9)	.388	45.6 (45.7)	46.9 (53.5)	43.5 (30.1)	.672
*High school diploma/GED*	67.2%	73.5%	59.3%	.366	73.5%	76.2%	69.2%	.962
*Any prior technical training*	37.7%	35.5%	40.7%	.865	35.3%	38.1%	30.8%	.948
*Past 3-year typical employment pattern*				.333				.432
Full-time/Part-time	27.9%	35.3%	18.5%		35.3%	28.6%	46.2%	
Retired/Disabled	31.1%	26.5%	37.0%		26.5%	33.3%	15.4%	
Unemployed	41.0%	38.2%	44.4%		38.2%	38.1%	38.5%	
*Years at longest-held full-time job*	7.0 (6.5)	7.0 (6.7)	7.0 (6.4)	.895	7.0 (6.7)	6.8 (7.5)	7.3 (5.5)	.381
*Skilled worker*^c^	29.5%	35.3%	22.2%	.407	35.3%	38.1%	30.8%	.727
*Any paid workdays, past month*	8.2%	8.88%	7.4%	.841	8.8%	9.5%	7.7%	1.00
*Past-month income (USD)*								
Employment income^d^	$7.7 (26.9)	$8.6 (29.2)	$6.6 (21.2)	.831	$8.6 (29.2)	$7.5 (23.6)	$10.4 (37.4)	.972
Non-employment income^e^	$484 (351)	$410 (336)	$577 (353)	.072	$410 (336)	$420 (335)	$394 (349)	.649
*Opioid agonist treatment, past month*								
Any	96.7%	100.0%	92.6%	.374	100.0%	100.0%	100.0%	–
Days	27.6 (8.0)	28.9 (5.8)	26.5 (9.6)	.059	28.6 (6.3)	29.4 (4.4)	27.9 (7.8)	.266
**Opioid use**								
*Lifetime*								
Heroin	100.0%	100.0%	100.0%	–	100.0%	100.0%	100.0%	–
Prescription opioids	31.1%	35.3%	25.9%	.613	35.3%	42.9%	23.1%	.292
*Past 30-days*								
Heroin	60.7%	58.8%	63.0%	.948	58.8%	57.1%	61.5%	1.00
Prescription opioids	21.3%	23.5%	18.5%	.873	23.5%	14.3%	38.5%	.211
**Cocaine use**								
*Lifetime*	78.7%	79.4%	77.8%	1.00	79.4%	71.4%	92.3%	.210
*Past 30-days*	57.4%	64.7%	48.1%	.299	64.7%	61.9%	69.2%	.727

a *p*-values calculated using *chi*-square, Fishers exact test, and 2-tailed Mann-Whitney *U* tests; all continuous variables are presented as means and standard deviations.

b Total calculated for those who were ever incarcerated: *n* = 52 for "Total enrolled" and *n* = 30 for "Completers."

c Hollingshead index of 7 vocational categories which were dichotomized to skilled vs. semi-skilled/unskilled.

d Employment income consisted of income from paid wages from taxed work as well as “under-the-table” untaxed work.

e Non-employment income consisted of income from unemployment benefits, welfare (e.g., cash assistance, food stamps), pensions/retirement disbursements, Social Security Insurance Disability benefits, money received from friends or family, and income generated through illicit activities.

f For the "Total enrolled" sample, opioid agonist treatment consisted of methadone treatment (90.2%, *n* = 55) and buprenorphine treatment (6.6%, *n* = 4); for the "Completers," opioid agonist treatment consisted of methadone treatment (94.1%, *n* = 32) and buprenorphine treatment (5.9%, *n* = 2).

### 3.2 Compliance with QOL questionnaires

Of the 67 participants enrolled into the Workplace Study between May 2017 and July 2018 (see Holtyn et al., 2020 for a CONSORT diagram of the Workplace Study), 61 (91.0%) responded to at least one QOL questionnaire. Of the 6 who did not: 1 participant received Delight Me training but provided no responses, and 5 participants did not receive training because they had already withdrawn from the Workplace Study (*n* = 2), ceased to attend the Therapeutic Workplace and were lost to follow-up (*n* = 2), or lost the smartphone (*n* = 1). Among those 61 participants, compliance with the QOL questionnaires was good: participants responded to 84.6% ± 2.5% (mean ± SEM) of their daily questionnaires and 87.0% ± 2.4% of their weekly questionnaires. Also, among those 61 participants, 11 (18.0%) used the Delight Me app to assign themselves additional daily and weekly self-monitoring or self-improvement tasks (e.g., “learn new words daily” or “make life better for me and my family”), separately from the QOL questionnaires they were assigned by the study.

Although participants’ remuneration depended only on their completing a majority of the QOL assessments each week, our manual data reviews and compliance meetings with participants suggested they predominantly responded in an “all-or-none" fashion (i.e., they completed 0/2 or 2/2 questions in the daily questionnaire and 0/30 or 30/30 questions in the weekly questionnaire). Inspection of the raw data supported this impression, and inspection of the partially completed questionnaires did not clearly indicate consistently missing items (i.e., they were not abandoned at a similar midpoint, leaving the later questions systematically unanswered). If anything, participants occasionally omitted the 2 questions on sleep when completing the longer weekly questionnaire, perhaps reflecting confusion about the need to complete both sets of questions on those days.

As noted above, participants could not be discharged early from the study only for noncompliance with the QOL questionnaires; for those who were classified as QOL non-completers for subsequent analyses (*n* = 27): 9 were noncompliant with EMA data collection which also resulted in the termination of QOL data collection (7 DWG, 2 IWG), 8 damaged/lost the smartphone (5 DWG, 3 IWG), 7 ceased to attend the Therapeutic Workplace and were lost to follow-up (5 DWG, 2 IWG), and 3 voluntarily withdrew from smartphone-based data collection (2 DWG, 1 IWG).

### 3.3 Analysis 1: effects of access to paid work: randomization to work immediately vs. waitlist delay

**Fig. 1:**
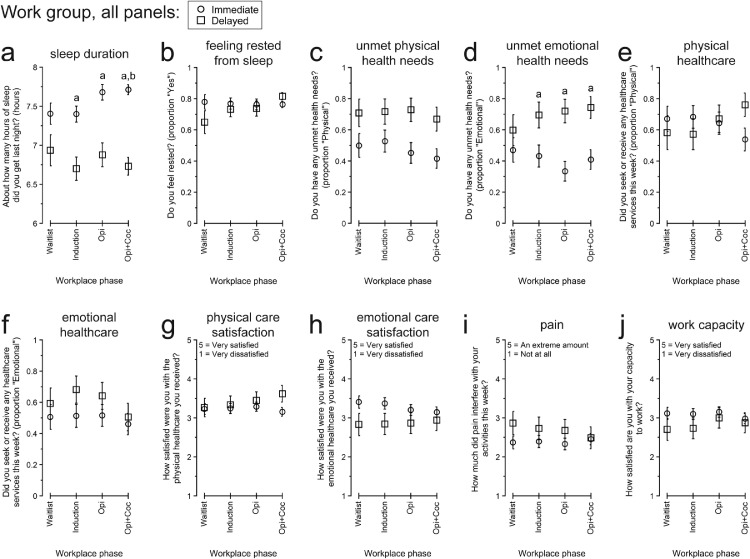
Participants’ responses to “Physical and emotional health” quality of life topics between the Immediate vs. Delayed Work Groups and across Therapeutic Workplace phases defined by participants’ access to paid work and the presence of abstinence-reinforcement contingencies. All data are presented as the least-squares means (± 1 standard error) from the multivariate model constructed for each endpoint. Within each panel, letters indicate statistically significant (*p* < .05) post-hoc pairwise differences: a, difference between groups within phase; b, different from Waitlist for Immediate Work Group. Opi, opiate urinalysis contingency; Opi+Coc, opiate and cocaine urinalysis contingencies.

**Fig. 2:**
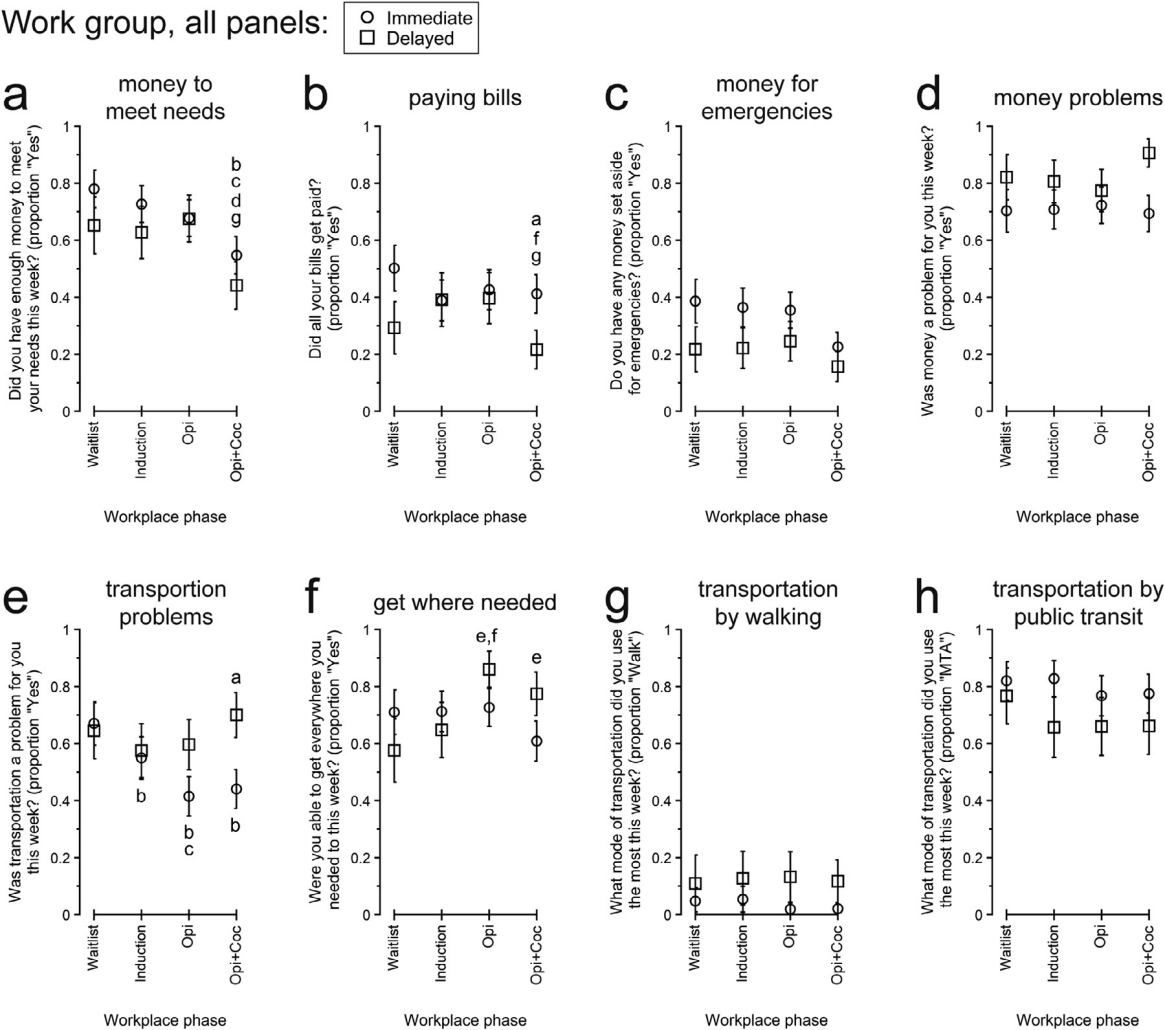
Participants’ responses to “Money” and “Transportation” quality of life topics between the Immediate vs. Delayed Work Groups and across Therapeutic Workplace phases defined by participants’ access to paid work and the presence of abstinence-reinforcement contingencies. All data are presented as the least-squares means (± 1 standard error) from the multivariate model constructed for each endpoint. Individual modes of transportation other than walking and Maryland Transit Administration (MTA) public transit are not presented because they were used infrequently, and the statistical models did not converge. Within each panel, letters indicate statistically significant (*p* < .05) post-hoc pairwise differences: a, difference between groups within phase; b, different from Waitlist for Immediate Work Group; c, different from Induction for Immediate Work Group; d, different from Opi for Immediate Work Group; e, different from Waitlist for Delayed Work Group; f, different from Induction for Delayed Work Group; g, different from Opi for Delayed Work Group. Opi, opiate urinalysis contingency; Opi+Coc, opiate and cocaine urinalysis contingencies.

**Fig. 3:**
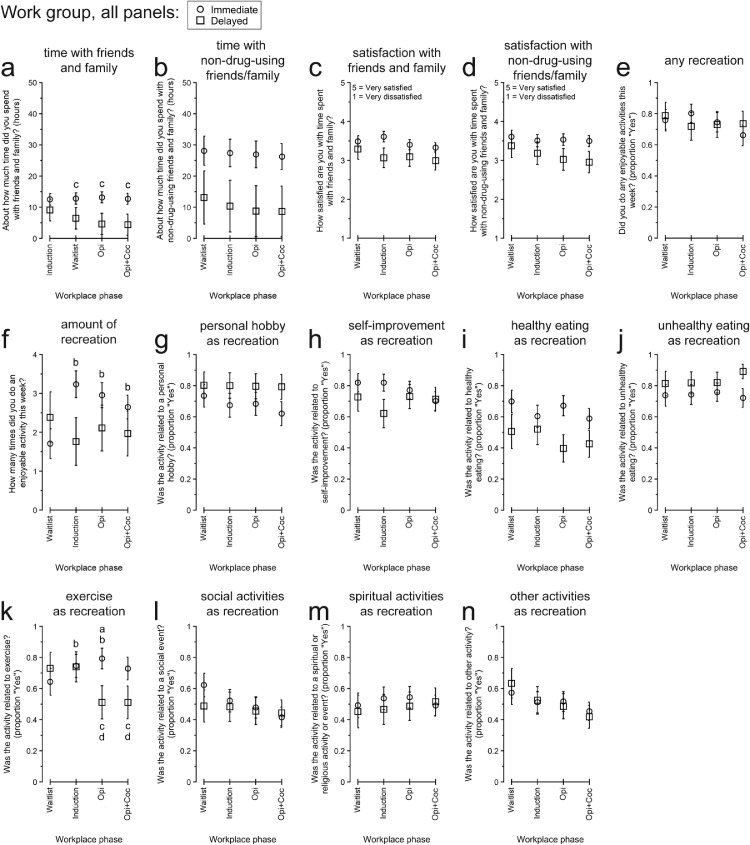
Participants’ responses to “Friends and family” and “Recreation” quality of life topics between the Immediate vs. Delayed Work Groups and across Therapeutic Workplace phases defined by participants’ access to paid work and the presence of abstinence-reinforcement contingencies. All data are presented as the least-squares means (± 1 standard error) from the multivariate model constructed for each endpoint. Within each panel, letters indicate statistically significant (*p* < .05) post-hoc pairwise differences: a, difference between groups within phase; b, different from Waitlist for Immediate Work Group; c, different from Waitlist for Delayed Work Group; d, different from Induction for Delayed Work Group. Opi, opiate urinalysis contingency; Opi+Coc, opiate and cocaine urinalysis contingencies.

Fig. 1, Fig. 2, Fig. 3 present participants’ responses to QOL questionnaires, separated by IWG vs. DWG, over the course of the Therapeutic Workplace phases that determined the groups’ access to paid work and the wage-resetting contingencies introduced to reinforce abstinence from opiates or opiates and cocaine. Table 3 presents the numerical details for the omnibus effects of Group and Phase for each endpoint.

**Table 3:** Omnibus analyses of quality of life differences between the Delayed Work Group (DWG) and Immediate Work Group (IWG) across Therapeutic Workplace phases determined by the groups’ access to paid work and urinalysis contingencies.

	Group				Phase					Group by Phase			
Quality of life Item	*df*	*F*	*p*_raw_	*p*_FDR_	*r*_effect_	*df*	*F*	*p*_raw_	*p*_FDR_	*df*	*F*	*p*_raw_	*p*_FDR_
**Physical and emotional health**													
Hours of sleep	***1,6***	***26.88***	***.0020***	***.0324***	***.90***	3,96	1.84	.1455	.3185	3,96	2.15	.0984	.2571
Feeling rested	1,28	1.01	.3234	.4501	.19	3,96	1.37	.2570	.4248	3,96	1.86	.1420	.3185
Unmet physical health needs	1,28	5.71	.0239	.1291	.41	3,96	1.03	.3813	.4752	3,96	0.52	.6695	.6695
Unmet emotional health needs	**1,28**	**8.34**	**.0074**	**.0749**	**.48**	3,96	1.08	.3627	.4728	3,96	2.36	.0766	.2386
Seeking or receiving physical healthcare	1,28	0.02	.8839	.8839	.03	3,94	0.18	.9074	.9074	3,94	3.53	.0177	.1103
Seeking or receiving emotional healthcare	1,28	1.06	.3109	.4468	.19	3,94	3.62	.0160	.1103	3,94	1.20	.3144	.4468
Satisfaction with physical healthcare	1,6	0.57	.4775	.5356	.29	3,88	0.70	.5554	.5768	3,88	2.17	.0977	.2571
Satisfaction with emotional healthcare	1,6	1.64	.2479	.4248	.46	3,95	0.41	.7496	.7496	3,95	1.21	.3093	.4468
Pain	1,6	0.78	.4103	.4960	.34	3,96	0.66	.5758	.5904	3,96	2.56	.0597	.2099
Capacity to work	1,6	0.73	.4259	.5000	.33	3,89	2.93	.0377	.1697	3,89	0.95	.4220	.5000
**Money**													
Having money to meet needs	1,28	0.82	.3736	.4728	.17	***3,95***	***7.68***	***.0001***	***.0041***	3,95	1.05	.3721	.4728
Paying bills	1,28	1.26	.2715	.4252	.21	3,95	3.43	.0202	.1169	***3,95***	***5.81***	***.0011***	***.0223***
Money set aside for emergencies	1,28	2.25	.1448	.3185	.27	3,95	3.80	.0127	.1029	3,95	0.16	.9262	.9262
Money being a problem	1,28	2.05	.1632	.3305	.26	3,95	1.40	.2468	.4248	3,95	2.53	.0622	.2099
**Transportation**													
Transportation being a problem	1,28	1.11	.3006	.4468	.20	**3,95**	**4.47**	**.0055**	**.0636**	3,95	2.43	.0699	.2265
Ability to get everywhere needed	1,28	0.14	.7076	.7076	.07	**3,95**	**4.62**	**.0046**	**.0621**	3,95	3.02	.0337	.1606
Most used transportation method: Walking	1,28	2.12	.1561	.3305	.27	3,95	0.35	.7867	.7867	3,95	0.35	.7882	.7882
Most used transportation method: MTA	1,28	0.97	.3324	.4501	.18	3,95	1.15	.3334	.4501	3,95	1.52	.2147	.3865
**Friends and family**													
Time spent with friends and family	1,6	2.56	.1610	.3305	.55	3,94	2.58	.0585	.2099	**3,94**	**3.98**	**.0102**	**.0918**
Time spent with friends and family who do not use drugs	1,6	2.95	.1367	.3185	.57	3,94	0.54	.6569	.6635	3,94	0.17	.9131	.9131
Satisfaction with time spent with friends and family	1,6	1.43	.2774	.4252	.44	3,95	1.60	.1943	.3747	3,95	2.81	.0435	.1762
Satisfaction with time spent with friends and family who do not use drugs	1,6	1.42	.2782	.4252	.44	3,93	1.97	.1240	.3044	3,93	0.73	.5347	.5651
**Recreation**													
Any enjoyable activities	1,28	0.00	.9944	.9944	.00	3,93	0.78	.5060	.5465	3,93	2.20	.0935	.2571
Number of enjoyable activities	1,6	0.70	.4351	.5035	.32	3,92	2.07	.1095	.2772	***3,92***	***9.89***	***<0.0001***	***.0041***
Hobby activities	1,28	1.30	.2640	.4252	.21	3,93	0.89	.4512	.5147	3,93	0.73	.5372	.5651
Self-improvement activities	1,28	0.86	.3618	.4728	.17	3,93	1.37	.2566	.4248	3,93	2.88	.0401	.1710
Healthy eating	1,28	3.06	.0912	.2571	.31	3,93	0.81	.4893	.5356	3,93	3.08	.0314	.1590
Unhealthy eating	1,28	1.65	.2097	.3680	.24	3,93	0.49	.6922	.6922	3,93	1.65	.1826	.3607
Exercising	1,28	0.71	.4059	.4960	.16	3,93	3.58	.0168	.1103	***3,93***	***7.46***	***.0002***	***.0054***
Social event	1,28	0.18	.6717	.6717	.08	3,93	1.54	.2097	.3860	3,93	0.82	.4842	.5356
Spiritual activities	1,28	0.12	.7318	.7318	.07	3,93	0.30	.8244	.8244	3,93	0.49	.6868	.6868
Other types of enjoyable activities	1,28	0.00	.9807	.9807	.00	3,94	2.61	.0560	.2099	3,94	0.24	.0870	.2571

Text of results is bolded where *p*_FDR_ < 0.10 and italicized where *p*_FDR_ < 0.05.

To ensure the included participants contacted all phases of the Therapeutic Workplace intervention, all analyses were conducted in the *n* = 34 study completers. All models included control terms for sex, race/ethnicity, vocational skill, and age. Models for previous-night hours of sleep and the amount of time spent with friends and family who do not use drugs also included a control term for astronomical season. Additional details are provided in the description of “Analysis 1” in the Methods section.

#### 3.3.1 Physical and emotional health

Fig. 1 presents participants’ responses to QOL items concerning physical and emotional health.

*Sleep*

Participants’ previous-night hours of sleep (Fig. 1a) differed significantly between groups, as indicated by the significant main effect of Group. By pairwise comparison, IWG reported sleeping more than DWG during Induction, *t*(96) = 3.65, *p* = .0036; Opi, *t*(96) = 4.16, *p* = .0006; and Opi+Coc, *t*(96) = 6.89, *p* < .0001. Within-group, IWG also reported sleeping more during Opi+Coc than Induction, *t*(96) = 2.94, *p* = .0369. Despite these differences in self-reported sleep amount, participants’ reports of feeling rested (Fig. 1b) did not differ significantly by Group or Phase.

*Unmet health needs*

IWG were numerically less likely than DWG to report unmet physical health needs throughout the study (Fig. 1c), although the main effect of Group was not significant after FDR correction. For emotional health (Fig. 1d), there was a trend for a main effect of Group. By pairwise comparison, IWG were significantly less likely than DWG to report having unmet emotional health needs during Induction, aOR (95% CI) = 0.334 (0.131, 0.855), *p* = .0227; Opi, aOR (95% CI) = 0.195 (0.078, 0.484), *p* = .0006; and Opi+Coc, aOR (95% CI) = 0.239 (0.101, 0.568), *p* = .0014.

*Seeking or receiving healthcare services*

Participants’ likelihood of seeking or receiving physical (Fig. 1e) or emotional (Fig. 1f) healthcare services did not differ significantly by Group or Phase. Likewise, their satisfaction with their physical (Fig. 1g) and emotional (Fig. 1h) healthcare did not differ significantly by Group or Phase.

*Pain interference and capacity to work*

Neither participants’ ratings of the extent to which pain interfered with their activities (Fig. 1i) nor of their satisfaction with their capacity to work (Fig. 1j) differed significantly by Group or Phase. Broadly, throughout the study, participants reported between “a little” and “a moderate amount” of interference with their activities from pain, and approximately neutral (“neither satisfied nor dissatisfied”) ratings of their capacity to work.

#### 3.3.2 Money

Figs. 2a-2d present participants’ responses to QOL items concerning money or personal finances.

*Having money to meet needs*

Participants’ likelihood of having enough money to meet their needs (Fig. 2a) differed significantly across Phases, but not by Group. Both IWG and DWG were more likely to have money to meet their needs during earlier phases than Opi+Coc. Compared to Opi+Coc, IWG were more likely to have money to meet their needs during Waitlist, aOR (95% CI) = 2.932 (1.131, 7.575), *p* = .0181; Induction, aOR (95% CI) = 2.201 (1.035, 4.679), *p* = .0294; and Opi, aOR (95% CI) = 1.736 (0.990, 3.044), *p* = .0379. DWG were more likely to have money to meet their needs during Opi than Opi+Coc, aOR (95% CI) = 2.636 (1.309, 5.307), *p* = .0019.

*Paying bills*

Participants’ likelihood of paying all their bills (Fig. 2b) differed across Phases, differently by Group, as indicated by the significant Group X Phase interaction. By pairwise comparison, IWG were more likely than DWG to pay all their bills during Opi+Coc, aOR (95% CI) = 2.535 (1.003, 6.410), *p* = .0493, with a trend for IWG to be more likely than DWG during Waitlist, aOR (95% CI) = 2.434 (0.846, 7.000), *p* = .0978. Within-group, compared to Opi+Coc, DWG were more likely to pay all their bills during Induction, aOR (95% CI) = 2.328 (0.988, 5.487), *p* = .0463, and Opi, aOR (95% CI) = 2.379 (1.266, 4.473), *p* = .0022. There was a trend for IWG to be less likely to pay all their bills during Induction than Waitlist, aOR (95% CI) = 0.631 (0.397, 1.003), *p* = .0524.

*Money set aside for emergencies*

Participants’ likelihood of having any money set aside for emergencies (Fig. 2c) did not differ significantly by Group or Phase. Throughout, participants were relatively unlikely to have emergency money.

*Money problems*

The likelihood that participants reported that money was a problem for them (Fig. 2d) did not differ significantly by Group or Phase. Money problems were reported frequently throughout the study.

#### 3.3.3 Transportation

Figs. 2e-2h present participants’ responses to QOL items concerning transportation.

*Transportation problems*

There was a trend for a main effect of Phase for the likelihood that transportation was a problem (Fig. 2e). Broadly, transportation problems decreased over time for IWG. Compared to Waitlist, IWG were less likely to report that transportation was a problem during Induction, aOR (95% CI) = 0.599 (0.363, 0.990), *p* = .0215; Opi, aOR (95% CI) = 0.349 (0.178, 0.684), *p* = .0003, and Opi+Coc, aOR (95% CI) = 0.387 (0.174, 0.862), *p* = .0095. IWG were also less likely to report that transportation was a problem during Opi than Induction, aOR (95% CI) = 0.582 (0.368, 0.919), *p* = .0095. During Opi+Coc, IWG were also less likely than DWG to report that transportation was a problem, aOR (95% CI) = 0.337 (0.136, 0.838), *p* = .0198.

*Ability to get everywhere needed*

There was a trend for a main effect of Phase for the likelihood that participants were able to get everywhere they needed (Fig. 2f). By pairwise comparison, DWG were more likely to be able to get everywhere needed during Opi than Waitlist, aOR (95% CI) = 4.545 (1.384, 14.924), *p* = .0053, and Induction, aOR (95% CI) = 3.355 (1.293, 8.695), *p* = .0053.

*Most used modes of transportation*

Models were constructed for the likelihood that participants used walking (Fig. 2g) or the MTA (i.e., Maryland Transit Administration public transit, Fig. 2h) as their most used mode of transportation. The other modes of transportation listed as response options were endorsed rarely, and their statistical models did not converge. For both walking and the MTA, all omnibus effects of group and phase and all pairwise comparisons were not significant.

#### 3.3.4 Friends and family

Figs. 3a-3d present participants’ responses to QOL items related to friends and family.

*Time spent with friends and family*

For the amount of time participants spent with friends and family (Fig. 3a), there was a trend for a Group X Phase interaction. Overall, DWG (but not IWG) spent numerically less time with friends and family across phases. By pairwise comparison to Waitlist, DWG spent less time with friends and family during Induction, *t*(94) = 3.18, *p* = 0.0220; Opi, *t*(94) = 3.75, *p* = .0033; and Opi+Coc, *t*(94) = 3.30, *p* = .0154. The amount of time participants spent with friends and family who do not use drugs (Fig. 3b) did not differ significantly by Group or Phase. IWG spent numerically more time with friends and family who do not use drugs, although the variability was large for both groups.

*Satisfaction with time spent with friends and family*

Participants’ satisfaction with their time spent with friends and family (Fig. 3c), and with friends and family who do not use drugs (Fig. 3d), did not differ significantly by Group or Phase. In both cases, participants generally rated their satisfaction with their friends and family as neutral or slightly-above-neutral.

#### 3.3.5 Recreation/enjoyable non-drug activities

Figs. 3e-3n present participants’ responses to QOL items concerning recreation.

*Presence of enjoyable activities*

The likelihood that participants engaged in any enjoyable activities (Fig. 3e) did not differ significantly by Group or Phase, with participants frequently engaging in at least one enjoyable activity in the past week. For the number of enjoyable activities (Fig. 3f), only the Group X Phase interaction was significant. Numerically, IWG initially engaged in fewer, but then engaged in more, enjoyable activities than DWG. Within-group, compared to Waitlist, IWG engaged in more enjoyable activities during Induction, *t*(92) = 6.31, *p* < .0001; Opi, *t*(92) = 3.95, *p* = .0002; and Opi+Coc, *t*(92) = 2.64, *p* = .0098. There was also a trend for IWG to engage in more enjoyable activities during Induction than Opi+Coc, *t*(92) = 1.96, *p* = .0534, and for DWG to engage in fewer enjoyable activities during Induction than Waitlist, *t*(92) = 1.94, *p* = .0560. Finally, between groups, there was a trend for IWG to engage in more enjoyable activates than DWG during Induction, *t*(92) = 1.94, *p* = .0554.

*Types of enjoyable non-drug activities*

Figs. 3g-3n present the likelihood that participants engaged in specific types of enjoyable non-drug activity. There were significant differences only for exercising (Fig. 3k), which varied across phases, differently for the groups, as indicated by the significant Group X Phase interaction. Generally, DWG were numerically less likely to exercise, but only in the latter half of the study. Within groups, compared to Waitlist, IWG were more likely to exercise during Induction, aOR (95% CI) = 1.623 (1.036, 2.541), *p* = .0225, and Opi, aOR (95% CI) = 2.124 (1.126, 4.006), *p* = .0112. Between groups, IWG were more likely to exercise than DWG during Opi, aOR (95% CI) = 3.663 (1.169, 11.484), *p* = .0264, with a trend during Opi+Coc, aOR (95% CI) = 2.569 (0.867, 7.608), *p* = .0877.

### 3.4 Analysis 2: associations between verified drug abstinence and QOL

**Fig. 4:**
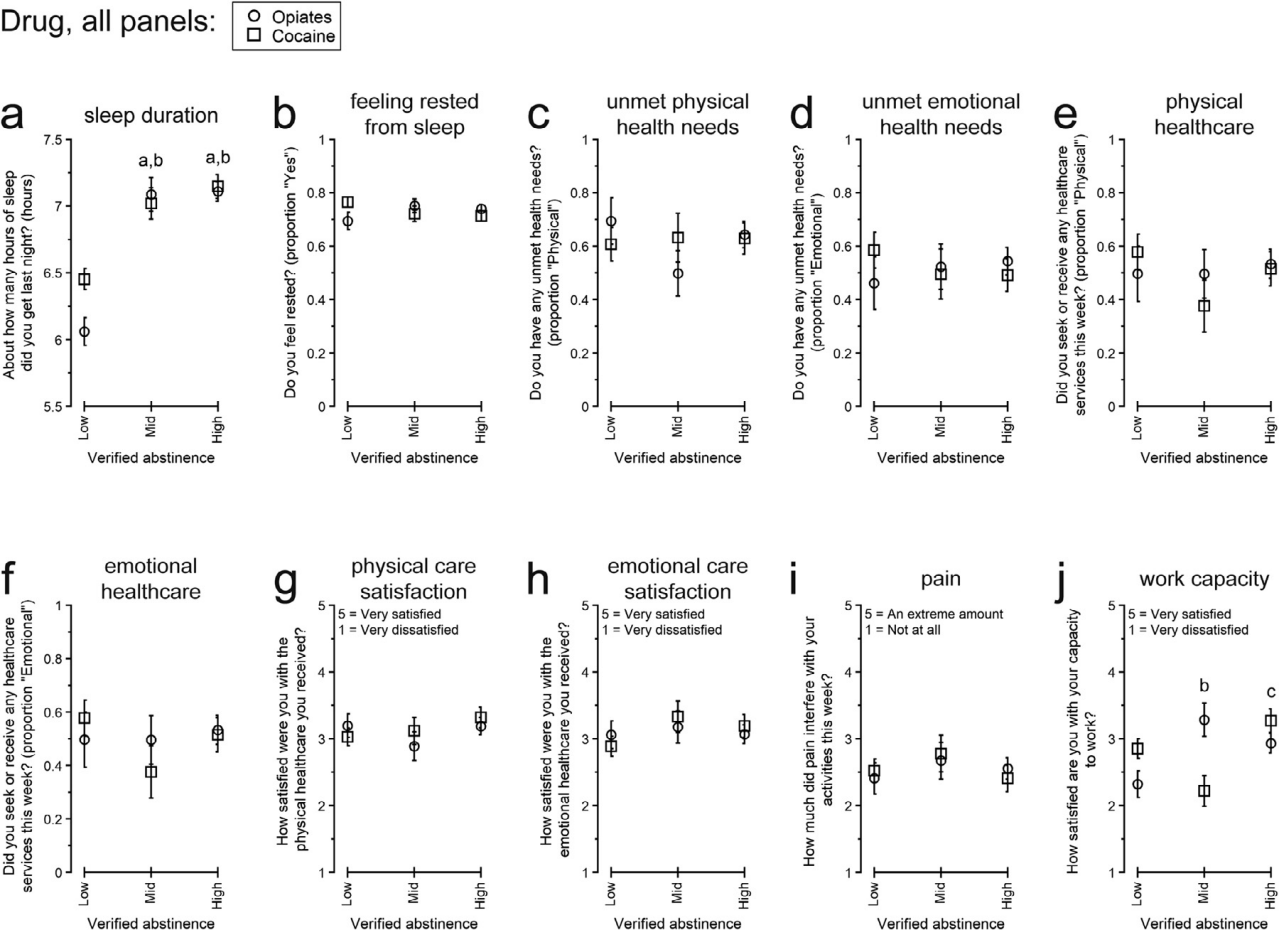
Responses to “Physical and emotional health” quality of life topics among participants with different overall amounts of urinalysis-verified drug abstinence: Low, Intermediate (Mid), or High. All data are presented as the least-squares means (± 1 standard error) from the multivariate model constructed for each endpoint. Within each panel, letters indicate statistically significant (*p* < .05) post-hoc pairwise differences: a, different from Low for opiate abstinence; b, different from Low for cocaine abstinence; c, different from Mid for cocaine abstinence.

**Fig. 5:**
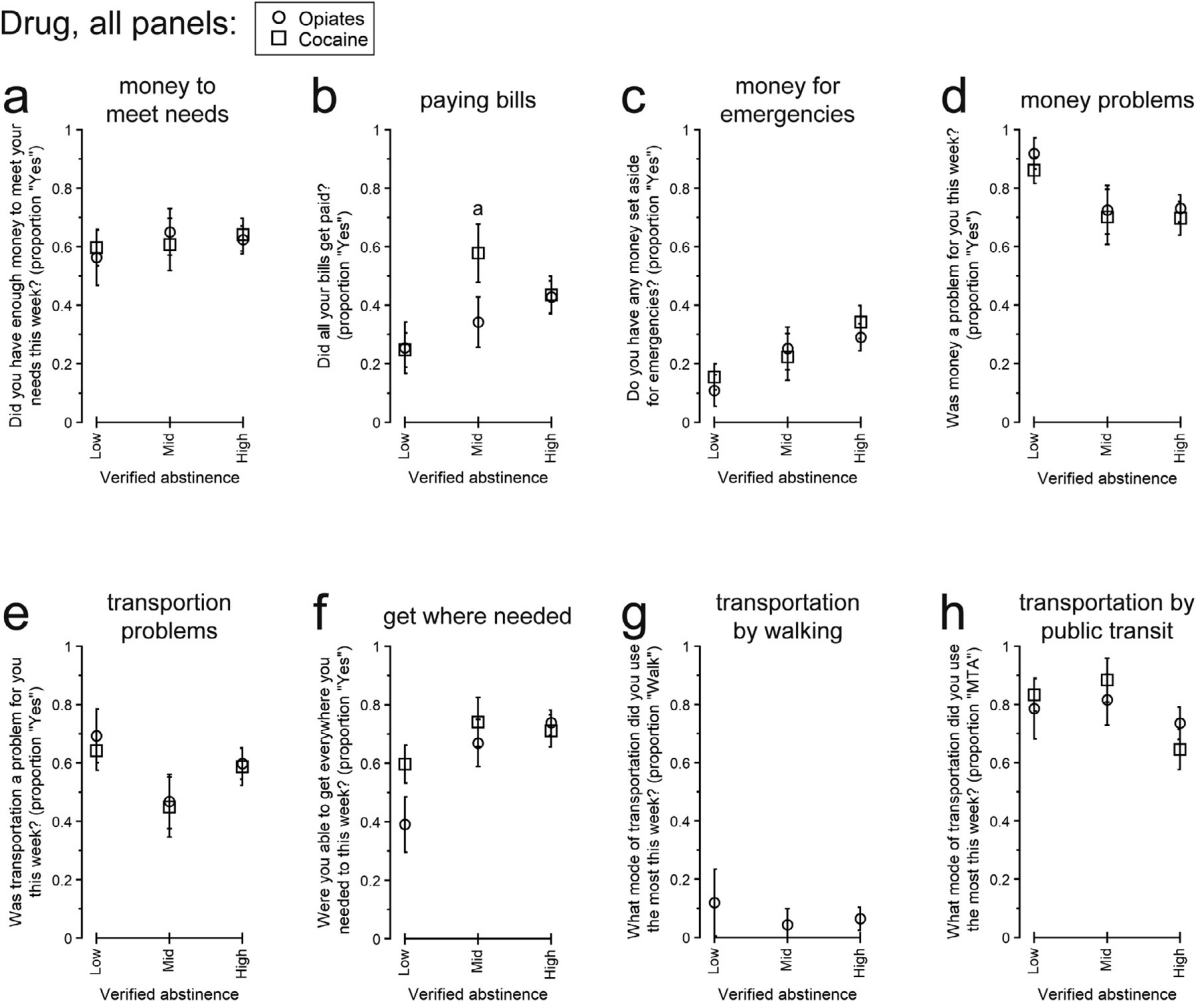
Responses to “Money” and “Transportation” quality of life topics among participants with different overall amounts of urinalysis-verified drug abstinence: Low, Intermediate (Mid), or High. All data are presented as the least-squares means (± 1 standard error) from the multivariate model constructed for each endpoint. Individual modes of transportation other than walking and Maryland Transit Administration (MTA) public transit are not presented because they were used infrequently, and the statistical models did not converge. For transportation by walking (panel g), only opiate abstinence is presented because the statistical model for cocaine abstinence did not converge. Within each panel, letters indicate statistically significant (*p* < .05) post-hoc pairwise differences: a, different from Low for cocaine abstinence.

**Fig. 6:**
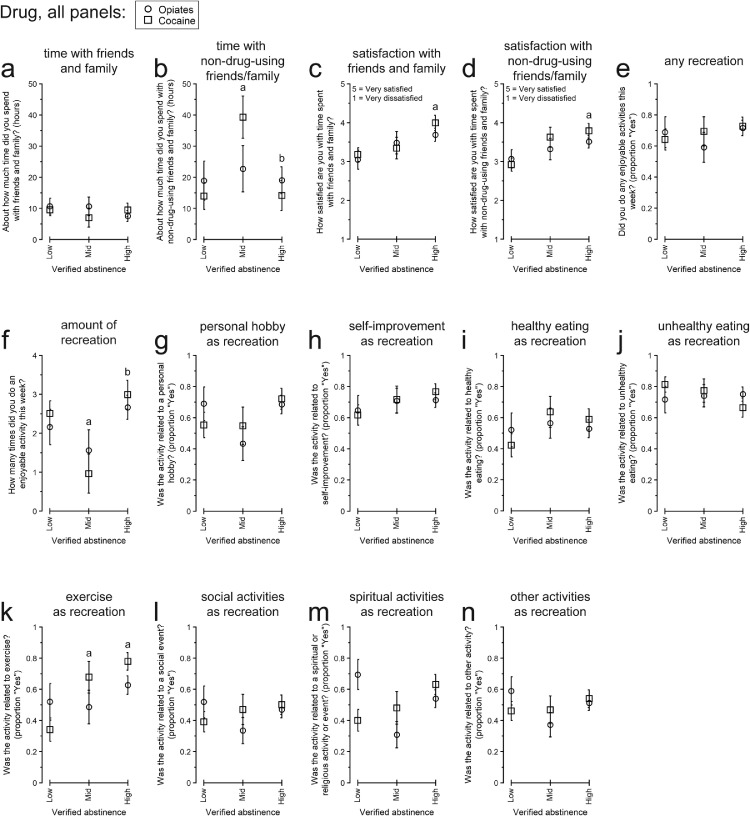
Responses to “Friends and family” and “Recreation” quality of life topics among participants with different overall amounts of urinalysis-verified drug abstinence: Low, Intermediate (Mid), or High. All data are presented as the least-squares means (± 1 standard error) from the multivariate model constructed for each endpoint. Within each panel, letters indicate statistically significant (*p* < .05) post-hoc pairwise differences: a, difference from Low for cocaine abstinence; b, different from Mid for cocaine abstinence.

Fig. 4, Fig. 5, Fig. 6 present participants’ responses to QOL questionnaires separated by overall rates of verified abstinence from opiates and cocaine. Table 4 presents the numerical details for the omnibus effect of Abstinence for each endpoint.

**Table 4:** Omnibus analyses of quality of life differences by participants’ urinalysis-verified abstinence from opiates and cocaine.

	Opiate abstinence				Cocaine abstinence			
Quality of life Item	*df*	*F*	*p*_raw_	*p*_FDR_	*df*	*F*	*p*_raw_	*p*_FDR_
**Physical and emotional health**								
Hours of sleep	***2,21***	***34.06***	***<0.0001***	***.0032***	***2,21***	***18.76***	***<0.0001***	***.0021***
Feeling rested	2,53	1.10	.3392	.6784	2,53	2.09	.1338	.2051
Unmet physical health needs	2,20	0.29	.7549	.9389	2,20	0.55	.5835	.5835
Unmet emotional health needs	2,52	1.57	.2169	.5995	2,52	0.04	.9588	.9588
Seeking or receiving physical healthcare	2,52	0.29	.7474	.9389	2,52	0.64	.5314	.5314
Seeking or receiving emotional healthcare	2,52	2.02	.1423	.5963	2,52	0.94	.3955	.3955
Satisfaction with physical healthcare	2,52	0.10	.9052	.9389	2,52	1.50	.2317	.2780
Satisfaction with emotional healthcare	2,18	0.78	.4741	.7712	2,18	1.07	.3627	.3627
Pain	2,20	0.08	.9192	.9389	2,20	1.43	.2633	.2780
Capacity to work	2,18	4.99	.0189	.2016	***2,18***	***6.54***	***.0073***	***.0441***
**Money**								
Having money to meet needs	2,52	0.27	.7656	.9389	2,52	0.14	.8658	.8658
Paying bills	2,52	1.43	.2486	.5995	***2,52***	***4.66***	***.0138***	***.0509***
Money set aside for emergencies	2,52	2.09	.1335	.5963	2,52	3.23	.0475	.1108
Money being a problem	2,52	1.85	.1677	.5963	2,52	2.50	.0923	.1615
**Transportation**								
Transportation being a problem	2,52	1.43	.2486	.5995	2,52	1.34	.2705	.2780
Ability to get everywhere needed	2,51	5.20	.0088	.1408	2,51	1.31	.2780	.2780
Most used transportation method: Walking	2,51	0.31	.7334	.9389	2,51	2.86	.0666	.1399
Most used transportation method: MTA	2,51	0.18	.8337	.9389	–	–	–	–
**Friends and family**								
Time spent with friends and family	2,20	0.57	.5747	.8757	2,20	0.25	.7826	.7826
Time spent with friends and family who do not use drugs	2,20	0.10	.9094	.9389	**2,20**	**4.87**	**.0190**	**.0509**
Satisfaction with time spent with friends and family	2,20	2.03	.1576	.5963	**2,20**	**4.84**	**.0194**	**.0509**
Satisfaction with time spent with friends and family who do not use drugs	2,20	1.04	.3713	.6989	***2,20***	***6.12***	***.0084***	***.0441***
**Recreation**								
Any enjoyable activities	2,52	0.74	.4820	.7712	2,52	0.44	.6440	.6440
Number of enjoyable activities	2,20	1.43	.2623	.5995	**2,20**	**5.02**	**.0171**	**.0509**
Hobby activities	2,52	2.28	.1119	.5963	2,52	1.46	.2408	.2780
Self-improvement activities	2,52	0.21	.8096	.9389	2,52	1.63	.2058	.2701
Healthy eating	2,52	0.06	.9389	.9389	2,52	2.07	.1367	.2051
Unhealthy eating	2,52	0.08	.9238	.9389	2,52	1.98	.1486	.2080
Exercising	2,52	0.88	.4189	.7447	***2,52***	***8.50***	***.0006***	***.0063***
Social event	2,52	1.14	.3267	.6784	2,52	0.76	.4727	.4727
Spiritual activities	2,52	3.85	.0276	.2208	2,52	2.71	.0758	.1447
Other types of enjoyable activities	2,52	1.72	.1899	.5995	2,52	0.52	.5994	.5994

Text of results is bolded where *p*_FDR_ < 0.10 and italicized where *p*_FDR_ < 0.05.

All analyses were conducted in the *n* = 61 participants who provided any QOL data. All models included control terms for sex, race/ethnicity, vocational skill, age, group (Immediate Work Group vs. Delayed Work Group), and Therapeutic Workplace phase (as determined by the groups’ access to paid work and the presence of urinalysis contingencies). Additional details are provided in the description of “Analysis 2″ in the Methods section.

#### 3.4.1 Physical and emotional health

Fig. 4 presents participants’ responses to QOL items concerning physical and emotional health. There were significant differences only for participants’ previous-night hours of sleep (Fig. 4a) and satisfaction with their capacity to work (Fig. 4j).

Participants’ hours of sleep differed by their opiate abstinence and cocaine abstinence. For both drugs, those with Intermediate Abstinence and High Abstinence reported more sleep than those with Low Abstinence: Opiate Low vs. Opiate Intermediate, *t*(21) = 6.31, *p* < .0001; Opiate Low vs. Opiate High, *t*(21) = 7.77, *p* < .0001; Cocaine Low vs. Cocaine Intermediate, *t*(21) = 3.76, *p* = .0064; Cocaine Low vs. Cocaine High, *t*(21) = 5.95, *p* < .0001.

Participants’ satisfaction with their capacity to work differed by their cocaine abstinence. Participants with Intermediate Abstinence reported lower satisfaction than those with Low Abstinence, *t*(18) = 2.15, *p* = .0454, or High Abstinence, *t*(18) = 3.58, *p* = .0022. There was also a pairwise trend for those with High Abstinence to report greater satisfaction than those with Low Abstinence, *t*(18) = 1.86, *p* = .0792.

#### 3.4.2 Money

Figs. 5a-5d present participants’ responses to QOL items concerning money or personal finances. Among these, there was only an omnibus trend for participants’ likelihood of paying all their bills to differ by cocaine abstinence. By pairwise comparison, participants with Intermediate Abstinence were more likely to pay their bills than those with Low Abstinence, aOR (95% CI) = 4.166 (1.245, 13.888), *p* = .0154. There was also a pairwise trend for participants with High Abstinence to be more likely to pay all their bills than those with Low Abstinence, aOR (95% CI) = 2.341 (0.870, 6.289), *p* = .0765.

#### 3.4.3 Transportation

Figs. 5e-5h present participants’ responses to QOL items concerning transportation. Cocaine abstinence results are not reported for walking as the most used transportation method because the model did not converge. For all other endpoints, there were no significant differences by Abstinence after False Discovery Rate correction.

#### 3.4.4 Friends and family

Figs. 6a-6d present participants’ responses to QOL items related to friends and family. Differences were observed for the amount of time spent with friends and family who do not use drugs (Fig. 6b), satisfaction with time spent with friends and family (Fig. 6c), and satisfaction with time spent with friends and family who do not use drugs (Fig. 6d).

Considering friends and family who do not use drugs, for the omnibus effects, there was a trend for a difference by cocaine abstinence for the amount of time spent and a significant difference by cocaine abstinence for satisfaction with that time. By pairwise comparison, participants with Intermediate Abstinence spent more time with friends and family who do not use drugs than those with Low Abstinence, *t*(20) = 2.90, *p* = .0178, and High Abstinence, *t*(20) = 2.89, *p* = .0182. Compared to participants with Low Abstinence, those with High Abstinence reported greater satisfaction with time spent with friends and family who do not use drugs, *t*(20) = 3.40, *p* = .0058. There was also a pairwise trend for greater satisfaction between those with Intermediate Abstinence vs. Low Abstinence, *t*(20) = 2.10, *p* = .0966.

Similar to friends and family who do not use drugs, there was a trend for an omnibus difference for participants’ satisfaction with time spent with friends and family, with significantly greater satisfaction between those with High Abstinence vs. Low Abstinence by pairwise comparison, *t*(20) = 3.02, *p* = .0136.

#### 3.4.5 Recreation/enjoyable non-drug activities

Figs. 6e-6n present participants’ responses to QOL items concerning recreation. There were significant differences only in the number of enjoyable activities done (Fig. 6f) and in the likelihood of exercising (Fig. 6k).

There was a trend for an omnibus effect of cocaine abstinence for the number of enjoyable activities done. Participants with Intermediate Abstinence engaged in fewer enjoyable activities than those with Low Abstinence, *t*(20) = 2.38, *p* = .0272, and High Abstinence, *t*(20) = 3.16, *p* = .0049.

Participants’ likelihood of exercising differed significantly by their cocaine abstinence. Compared to those with Low Abstinence, participants with Intermediate Abstinence, aOR (95% CI) = 4.065 (1.085, 15.151), *p* = .0225, or High Abstinence, aOR (95% CI) = 6.802 (2.066, 22.727), *p* = .0006, were more likely to exercise.

### 3.5 Analysis 3: exploratory nonmetric multidimensional scaling QOL items

**Fig. 7:**
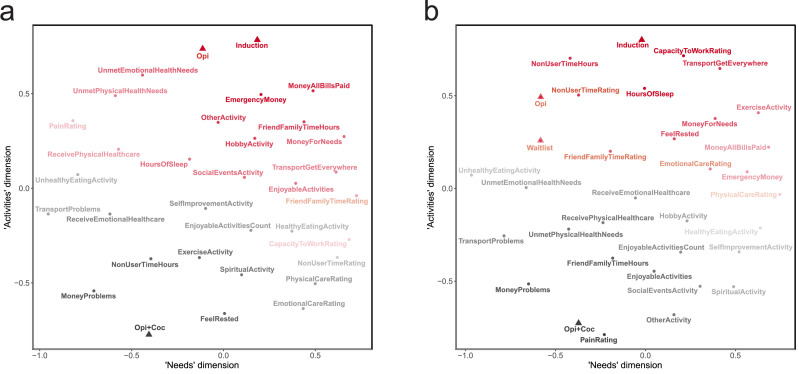
Arrangement of all quality of life items in two dimensions by nonmetric multidimensional scaling. a) responses of Immediate Work Group, b) responses of Delayed Work Group. Distances were determined as 1 minus the Spearman rank correlation coefficient for each pair of items: items with stronger correlations are closer to one another in space. The triangular points represent the Therapeutic Workplace phases. The color of each item indicates whether its correlational distance is closer to the Induction phase (dark red) or closer to the Opi+Coc phase (dark gray), and the shading is darker as a function of how closely the item is associated with the phase. The axis labels (*x*-axis as “Needs” and *y*-axis as “Activities”) represent our interpretation of the two dimensions made from inspection of the arrangement of the items.

The exploratory nonmetric multidimensional scaling results are presented as scattergrams (Fig. 7) depicting the similarities among QOL items in two dimensions. Having established in Analyses 1 and 2 when each QOL endpoint differed individually (i.e., was “better” or “worse” in-and-of itself), this analysis focuses on the overall pattern of similarities among participants’ responses to all QOL items at once to reveal associations both within and across the different QOL domains into which we had organized the items in writing the questionnaire. Patterns were similar for IWG (Fig. 7a) and DWG (Fig. 7b), and so we will consider them together. The triangular points represent the Therapeutic Workplace phases, with the greatest distance (i.e., indicating dissimilarity) between the Induction phase and the Opi+Coc phase and the other phases closer to Induction at the top of the figure than Opi+Coc at the bottom. The color and shading of each item represent its correlational distance from the Induction phase or the Opi+Coc phase: items more closely related to Induction are red and items more closely related to Opi+Coc are gray; shading is darker as a function of this closeness. To summarize the overall arrangement of items, we interpret the horizontal dimension as representing a continuum of needs related to health, finances, and transportation, with unsatisfied needs to the left (e.g., ongoing transportation problems, money problems, and pain) and satisfied needs to the right (e.g., having money for one's needs, being able to get everywhere needed, and satisfaction with healthcare that was received). The vertical dimension represents a continuum of activities differing by both orientation to others vs. self and recreation vs. obligation: socializing and recreational items towards the top and “self-care” items towards the bottom. Thus, summarizing across QOL domains and Workplace phases, responses during the final phase tended to differ considerably from responses during the other phases with respect to the Activities dimension. The first three phases (Induction, Waitlist, and Opi) were all associated with spending time with friends and family and having money to cover expenses. In contrast, Opi+Coc, the final phase, was more closely associated with resting, receiving care, and self-improvement. However, it was also associated with money problems, possibly related at least partly to lost study income (i.e., not becoming abstinent and experiencing wage resets). Compared to the strong differences among phases on the vertical dimension (Activities), differences among phases along the horizontal dimension (Needs) were small, but the location of the Induction phase to the right of other phases suggests the possibility that participants felt that their needs were being met to a greater extent during Induction.

## 4 Discussion

### 4.1 Changes in QOL domains between groups and across treatment phases

Participants experienced complex changes in several aspects of QOL over the course of the study. Considering possible benefits vs. tradeoffs associated with work, in Analysis 1 at least some participants (DWG and/or IWG) reported more sleep (Fig. 1a), improvements in transportation (Figs. 2e, 2f), and more non-drug enjoyable activities (Figs. 3f, 3k); however, they also reported greater money-related difficulties (Figs. 2a, 2b) and less time spent with friends and family (Fig. 3a). Whereas some of these differences were found across multiple phases (more enjoyable non-drug activities, less time spent with friends and family), others were selective for the Opi+Coc phase (sleep duration, money-related difficulties), suggesting the importance of abstinence reinforcement in addition to accessing paid work *per se* (cf. Silverman et al., 2016 on differences between contingent and non-contingent Therapeutic Workplace access).

The potential importance of abstinence reinforcement was also demonstrated in Analysis 3, with NMDS of all QOL items together highlighting differences between Opi+Coc and the earlier Therapeutic Workplace phases. Spending time with friends and family and meeting financial obligations (having money to meet needs, pay bills, and for emergencies) was more strongly associated with the earlier phases, whereas Opi+Coc was more strongly associated with “self-care” behaviors such as resting, receiving healthcare, and engaging in self-improvement activities for recreation. These results also highlight how different QOL elements representing different life domains may change together in response to particular life events or, for research participants, study events. For participants achieving abstinence, the later Therapeutic Workplace phases may reflect longer periods of decreased use and greater stabilization, thus enabling a shift towards self-investment and -care. However, participants experiencing wage resets due to positive/missing urine samples may have had reduced study-related income and, therefore, more money-related difficulties. This is an important consideration as economic insecurity can adversely influence QOL and SUD outcomes (Carlsen et al., 2019; Drobnič et al., 2010; Preston et al., 2018); however, the benefits of attempting to maximize participants’ money-related or money-dependent QOL should be balanced against the motivational effectiveness of the reinforcers in contingency management and the other QOL benefits associated with reduced drug use, which are discussed further below (Section 4.2).

Considering group differences, specifically, we expected differences between DWG and IWG during the Waitlist phase that would be reduced or eliminated once DWG could also access paid work. None of the group differences we found followed this pattern. Instead, we found differences during some or all of the phases (depending on the endpoint) when both groups had access to paid work. Notably, participants who were exposed to the waitlist condition (DWG) were consistently “worse off” than those who were not (IWG); this effect was surprisingly persistent over the subsequent phases of the study. DWG reported less sleep (Fig. 2a), greater unmet emotional healthcare needs (Fig. 2d), less having all their bills paid (Fig. 3b), greater transportation problems (Fig. 3e), and less exercise (Fig. 4k). As above, differences that emerged only once the abstinence-reinforcement contingencies were introduced (in bills paid, transportation problems, and exercising) may be related at least partly to the amount of time participants experienced work in the Therapeutic Workplace without contingencies before the contingencies were introduced (i.e., DWG had more difficulty adapting to work with the contingencies because they had less experience working/following the other Therapeutic Workplace requirements beforehand). These differences, as well as those that were found during Induction, may also be related to the use of a waitlist itself.

Although waitlist control groups have been commonly used to study behavioral/psychotherapeutic interventions for a variety of conditions, there is growing recognition that placement on a waitlist itself may significantly affect participants, i.e., that waiting for future treatment is not equivalent to no treatment or natural historical observation alone (e.g., Cunningham et al., 2013; Furukawa et al., 2014; Hart et al., 2008; Mohr et al., 2009; Patterson et al., 2016; Studacher et al. 2017). Knowing treatment is upcoming, participants’ may inhibit their own pro-health behaviors (Mohr et al., 2009; Patterson et al., 2016; cf. Cunningham et al., 2013), and participants may also develop different beliefs or expectations while waiting that could change their experience of their condition and/or its treatment (Cunningham et al., 2013; Mohr et al., 2009; Patterson et al., 2016; Whitehead 2004). Although these changes could sometimes be beneficial (Hart et al., 2008; Studacher et al. 2017), they seem more likely to be detrimental, compared with either immediate treatment-entry or a person's “natural recovery processes” (Harris and Miller 1990; Patterson et al., 2016). Waitlist-related negative moods (e.g., worry about not being able actually to receive treatment, disappointment at having to wait for a desired treatment) may also exacerbate participants’ symptoms/symptom reports (Cunningham et al., 2013; Patterson et al., 2016). Ultimately, possible waitlist effects are concerning generally; however, more work is needed to understand how they may impact people with SUDs in particular (for people who drink alcohol, see Cunningham et al., 2013; Gajecki et al., 2017; Kypri et al., 2015), and it is important to recognize that each of the various comparison conditions/groups used to study behavioral/psychotherapeutic interventions has its own strengths and weaknesses and potential threats to validity (Hart et al., 2008; Mohr et al., 2009; Patterson et al., 2016).

### 4.2 Abstinence and QOL

Although debate about the importance of total abstinence is ongoing (e.g., Panlilio et al., 2020), decreasing drug use is an important goal in SUD interventions generally (Best et al., 2013; Dennis et al., 2020; Donovan et al., 2012; Kelly et al., 2018; Silverman et al., 2001; Vanderplasschen et al., 2013). We found associations between greater abstinence and better QOL in several domains: sleep duration (Fig. 4a), paying bills (Fig. 5b), spending more time with friends and family that do not use drugs (Fig. 6b), satisfaction with time spent with friends and family (Fig. 6c), and exercising (Fig. 6k). Some of these findings are consistent with previous work (e.g., drug use associated with less sleep in daily life, Bertz et al., 2019). However, there were several surprising nonlinear relationships between cocaine abstinence level and QOL: time spent with friends and family (Fig. 6b), engaging in enjoyable activities (Fig. 6f), and satisfaction with capacity to work (Fig. 4j). We interpret these differences with caution, considering the relatively small sample size of the study, but they could point to potentially important considerations for those with moderated drug use vs. complete (or near-complete) abstinence and the use of QOL to measure treatment response (or recovery) (Witkiewitz et al., 2019, 2021; Kelly and Bergman, 2021).

Considering the particular QOL endpoints with nonlinear relationships with abstinence, participants with Intermediate cocaine abstinence reported spending more time with friends and family who did not use drugs compared to both the Low Abstinence group and the High Abstinence group (cf. MacDonald et al., 2004 on the social networks of people in recovery). Even so, the High Abstinence group, followed by the Intermediate Abstinence group, reported greater satisfaction with time spent with non-using friends and family compared to the Low Abstinence group, in keeping with some prior work (Mawson et al., 2015; Muller et al., 2017). These results may reflect the natural heterogeneity people with SUD experience and how friends/family could exert both positive and negative effects (Leach and Kranzler, 2013; Majer et al., 2002; Mowen and Visher, 2015; Preston et al., 2017; Wallace et al., 2016). Friends or family who do not use drugs may also still reside in areas characterized by drug-related cues (e.g., locations where drugs were previously used), and some in the High Abstinence group may have spent less time with them to avoid stress/cues, or for reasons which cannot otherwise be clearly discerned from our present data. Nonetheless, the greater satisfaction with time spent with non-drug-using friends and family reported by the High Abstinence group underscores the importance of relationships not characterized by drug use in helping to reinforce continued behavioral change (Bassuk et al., 2016; Bickel et al., 2014; MacDonald et al., 2004; Petry et al., 2001).

Participants with Intermediate Abstinence from cocaine also engaged in fewer enjoyable activities compared to those with both Low and High Abstinence. Although participants were instructed to report non-drug activities, it is possible that some participants also included drug use when reporting their enjoyable activities; if so, that could have inflated particularly the number of activities reported by the Low Abstinence group. For non-drug activities specifically, greater engagement with non-drug activities, including work-related activities, may help reduce drug use (Petry et al., 2001, 2014), and so this topic may deserve further attention to support those with difficulties accessing (or enjoying) non-drug recreation.

Satisfaction with capacity to work also showed nonlinear differences by cocaine abstinence (Fig. 5j), with less satisfaction reported by those with Intermediate Abstinence vs. both Low and High Abstinence. It is difficult to draw strong conclusions from this pattern, but it may be particularly important to understand this relationship to maximize the effectiveness of the Therapeutic Workplace and other vocational interventions for people with SUDs. Studies in other populations have found work capacity is associated with multiple characteristics of the individual, his/her clinical condition, and its treatment, as well as different elements of “capacity” (e.g., Chang et al., 2000; Gross et al., 2010; Mintz et al., 1992). Drug use, in particular, may reduce capacity to work by different mechanisms (e.g., physical impairments and cognitive impairments, Richardson and Epp 2016), which may change differently with amount of use. Withdrawal symptoms may also significantly impact work capacity as people decrease their drug use (cf. Richardson and Epp 2016); these effects may be more severe or salient (1) as people first begin to decrease their use or cycle between use and abstinence, both of which may have appeared in our analysis as Intermediate Abstinence, and (2) with cocaine use, as the OAT almost all participants were receiving would likely have ameliorated opioid withdrawal. These differences may also have interacted, at least temporarily (Holtyn and Silverman, 2016), with the wage-resetting procedures used in this study (e.g., as wage changes impacted work-related emotions and the affective elements of work capacity, cf. Mintz et al., 1992).

### 4.3 Limitations

As discussed above, the use of a waitlist may have produced different expectancies in DWG vs. IWG. Other comparison groups may have produced different results, but this possibility should be evaluated considering the rarity of the opportunity to control experimentally people's ability to begin working. We also used a novel questionnaire administered in a novel mobile format to assess QOL. We found this was feasible, with good participant compliance; however, we do not know how our results would compare to QOL assessed with commonly used instruments, such as the WHOQOL-BREF (Skevington et al., 2004). Although there are several QOL assessments that have been validated and widely used in the general population, validating QOL questionnaires specifically in people with SUD may present particular challenges (e.g., to establish sensitivity, Muller et al., 2016). Several measures have been proposed, which have been validated with different populations of people who use substances in different countries (e.g., Muller et al., 2016; Wan et al., 2011; Zubaran et al., 2012), but it is unclear how the validation done with laboratory-based administration may translate to mobile delivery. Having established here the feasibility of using our mobile web-based questionnaires, and having found participants’ responses differed within and across the domains we assessed in association with significant study events and treatment outcomes, future studies could include also versions of these other questionnaires for direct comparisons between instruments. The present study sample was also relatively small, particularly when only the completers and the different abstinence groups were considered. Overall, our analyses were feasible, and the use of multilevel models enabled appropriate handling of unbalanced aspects of the data, e.g., differences in participants’ compliance across weeks (Nich and Carroll, 1997); however, as noted when describing the specific analyses, several models did not converge due to a lack of participants endorsing particular responses (e.g., individual modes of transportation). We also were not able to include random intercepts in our models. Using fixed-intercept models, we were able to test for within-person associations while controlling for (though not specifically modeling) between-person differences (Snijders and Bosker, 2012). Replication in larger samples of people with SUDs, as well as workers with and without different health conditions, is needed. Finally, although compliance in our study was good, it is important to recognize that participants received training and ongoing support from study staff for the duration of data collection, and so different results may be obtained in other kinds of environments/treatment programs.

### 4.4 Clinical implications and conclusion

QOL domains, such as physical and mental health, receiving services, financial stability, transportation, social interaction and support, employment, and recreation are consonant with the idea of “recovery capital” (Best and Laudet, 2010; Cano et al., 2017; Cloud and Granfield, 2008; Groshkova et al., 2013; Kaskutas et al., 2014; Laudet et al., 2009; Laudet and White, 2010). Improvements in QOL may be important outcomes for SUD treatment themselves, as well as drivers of other drug-related outcomes of interest. This makes measurement of QOL during and after treatment an important area of continued focus for researchers and clinicians (Bray et al., 2017; Laudet, 2011; Logan et al., 2020; Mareva et al., 2016; Maremmani et al., 2007; Smith and Larson, 2003).

For contingency management interventions, specifically, there may be skepticism among treatment providers about financial incentives for abstinence improving QOL (Hartzler and Rabun 2013), and so it is notable that our findings add to the literature showing improvements in QOL during or after contingency management (Andrade et al., 2012; EÉk et al., 2020; Epstein et al., 2009; Petry et al., 2007). Beyond the specific differences between study groups and among phases described above, our QOL assessments also highlighted participants’ persistent needs or difficulties in multiple life areas (e.g. high overall rates of money problems, transportation problems, and healthcare needs). Participants did not receive individual clinical counseling during this study, but with such counseling QOL could be assessed by case workers or therapists and used to tailor treatment, training, or other support services to individual patients. Although we did not directly compare laboratory-based vs. mobile assessment of QOL, web apps, such as the Delight Me platform used here, could be particularly useful with their associated “clinician side” vs. “patient side” interfaces or dashboards for efficiently tracking and summarizing data. In contexts where healthcare professionals can easily obtain up-to-date QOL information on their patients, there is considerable potential for making QOL measurement more patient-centric and “user-generated,” based on what patients perceive as especially relevant to understanding their QOL (Alves et al., 2017; Brown and Ashford, 2019; Ruta et al., 1994). This may make treatment itself more precise, person-centered, and sensitive to change (Lasalvia et al., 2005).

## Role of funding source

This research was supported by the Intramural Research Program of the NIH, NIDA and by NIDA Grant R01DA037314. NIDA had no involvement in study design; in the collection, analysis and interpretation of data; in the writing of the report; and in the decision to submit the article for publication.

## Contributors

Jeremiah Bertz: Conceptualization, Project administration, Investigation, Data curation, Formal analysis, Visualization, Writing - Original draft. Kirsten Smith: Formal analysis, Writing - Original draft. Leigh Panlilio: Formal analysis, Visualization, Writing - Original draft. Samuel Stull: Investigation, Data curation, Writing - Review & editing. David Reamer: Investigation, Data curation. Marie-Louise Murville: Methodology, Writing - Review & editing. Michael Sullivan: Methodology, Software. August Holtyn: Conceptualization, Project administration, Investigation, Data curation, Writing - Review & editing. Forrest Toegel: Data curation, Writing - Review & editing. David Epstein: Conceptualization, Writing - Review & editing. Karran Phillips: Writing - Review & editing. Kenzie Preston: Conceptualization, Writing - Review & editing, Supervision, Funding acquisition.

## Declaration of Competing Interest

Marie-Louise Murville and Michael Sullivan declare financial interests in Delight Me Inc.: Marie-Louise Murville is the founder & CEO of Delight Me Inc., and Michael Sullivan is Vice President of Engineering of Delight Me Inc. All other authors have no interests to declare.

## Appendix Supplementary materials

Image, application 1
