# Supplementary material for: Quality of life during a randomized trial of a therapeutic-workplace intervention for opioid use disorder: Web-based mobile assessments reveal effects of drug abstinence and access to paid work
Source: Drug Alcohol Depend Rep. 2021 Dec 4;1:100011. doi: 10.1016/j.dadr.2021.100011 (PMC9948824; doi:10.1016/j.dadr.2021.100011)
Supplement: Supplementary file 1 [file mmc1.pdf]

Supplementary Figure 1: Schematic timeline of the study showing the Therapeutic Workplace access of participants randomized to the immediate work group (IWG) vs. delayed work group (DWG) and the introduction of wage-resetting contingencies for participants' opiate and cocaine urinalysis, which defined the study's experimental phases.

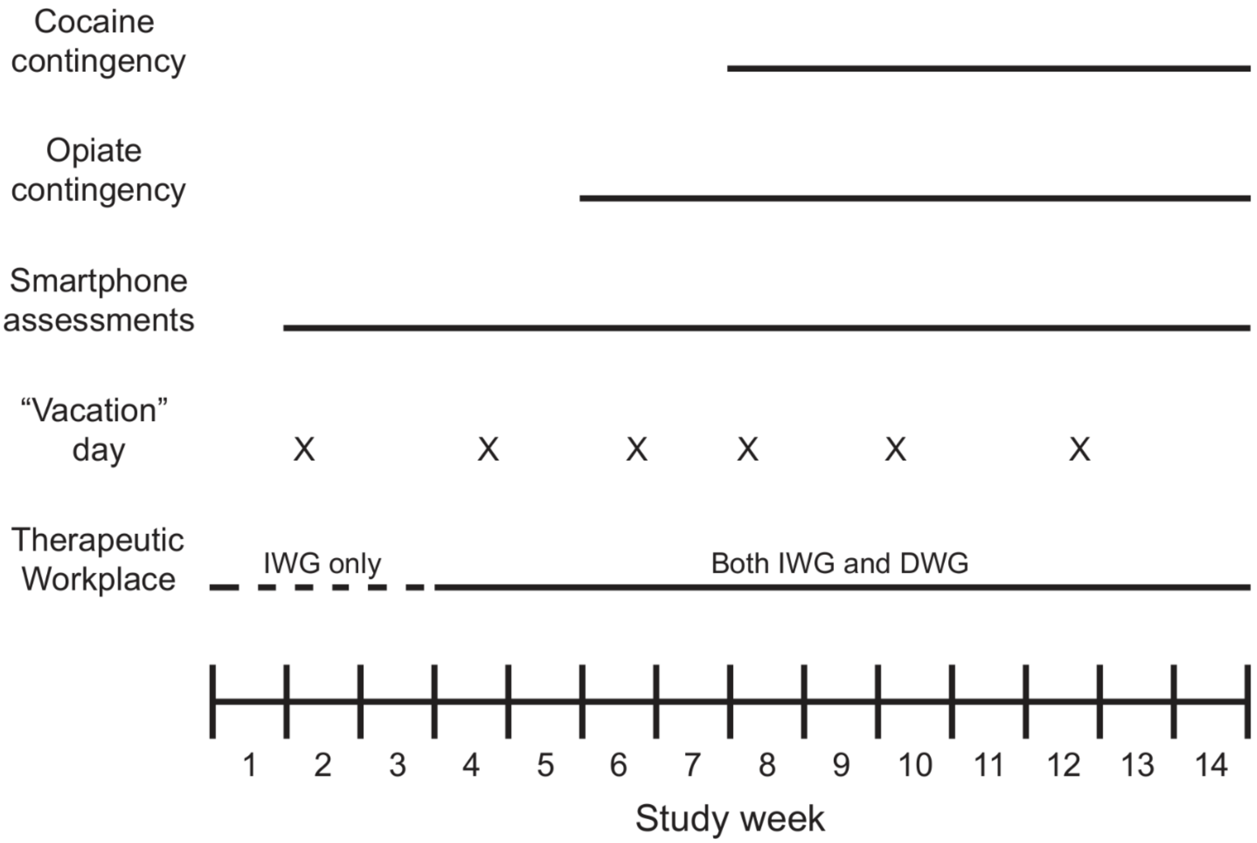

"Vacation" days refer to experimentally determined days when the Therapeutic Workplace was closed to all participants. These were not part of the analyses of participants' quality of life.
